# Millimeter-Level Plant Disease Detection From Aerial Photographs *via* Deep Learning and Crowdsourced Data

**DOI:** 10.3389/fpls.2019.01550

**Published:** 2019-12-12

**Authors:** Tyr Wiesner-Hanks, Harvey Wu, Ethan Stewart, Chad DeChant, Nicholas Kaczmar, Hod Lipson, Michael A. Gore, Rebecca J. Nelson

**Affiliations:** ^1^Plant Breeding and Genetics Section, School of Integrative Plant Science, Cornell University, Ithaca, NY, United States; ^2^Department of Computer Science, Columbia University, New York, NY, United States; ^3^Department of Mechanical Engineering and Institute of Data Science, Columbia University, New York, NY, United States; ^4^Plant Pathology and Plant-Microbe Biology Section, School of Integrative Plant Science, Cornell University, Ithaca, NY, United States

**Keywords:** phenotyping, unmanned aerial vehicles, plant disease, deep learning, machine learning, crowdsourcing

## Abstract

Computer vision models that can recognize plant diseases in the field would be valuable tools for disease management and resistance breeding. Generating enough data to train these models is difficult, however, since only trained experts can accurately identify symptoms. In this study, we describe and implement a two-step method for generating a large amount of high-quality training data with minimal expert input. First, experts located symptoms of northern leaf blight (NLB) in field images taken by unmanned aerial vehicles (UAVs), annotating them quickly at low resolution. Second, non-experts were asked to draw polygons around the identified diseased areas, producing high-resolution ground truths that were automatically screened based on agreement between multiple workers. We then used these crowdsourced data to train a convolutional neural network (CNN), feeding the output into a conditional random field (CRF) to segment images into lesion and non-lesion regions with accuracy of 0.9979 and F1 score of 0.7153. The CNN trained on crowdsourced data showed greatly improved spatial resolution compared to one trained on expert-generated data, despite using only one fifth as many expert annotations. The final model was able to accurately delineate lesions down to the millimeter level from UAV-collected images, the finest scale of aerial plant disease detection achieved to date. The two-step approach to generating training data is a promising method to streamline deep learning approaches for plant disease detection, and for complex plant phenotyping tasks in general.

## Introduction

Machine learning models for object detection require a large amount of training data, typically generated by humans. When the average person can identify the feature or object in question, such as a face, a stop sign, or an apple, these data can be generated through crowdsourcing, as was done for large datasets such as ImageNet (Deng et al., 2009) and Microsoft COCO (Lin et al., 2014). Even if the feature is unfamiliar to most people, crowdsourcing may be viable if the task is simple and the feature obvious. In a recent study on best practices for crowdsourcing plant feature annotation, Zhou et al. (2018) found that, with minimal instruction, anonymous online workers could accurately identify maize male flowers in images where they were clearly visible. Accurate identification of many plant features requires a certain level of expertise, however. If only a handful of human experts are qualified and willing to generate training data, the process takes much longer than if tasks could be reliably performed by hundreds or thousands of non-experts. This places a burden on those experts and creates a bottleneck in the model training process.

This dilemma has been addressed by many groups, particularly in the field of human medicine, wherein a model trained on low-quality data could endanger lives, but experts’ time is limited and expensive. Different circumstances allow for distinct solutions to the problem. For some tasks, such as interpreting X-ray radiographs, large amounts of training data are already generated and archived under normal protocols, and these data can be used as is without need for additional annotations (Gale et al., 2017). When untrained workers perform moderately well, but not quite on par with experts, their annotations can be used to train a “first pass” model that identifies regions of interest (Park et al., 2017), or one that performs only those tasks that non-experts can do well (Heim et al., 2018). Researchers might have access to a community of knowledgeable, enthusiastic amateurs, such as those who enjoy identification of birds (Van Horn et al., 2015) or aircraft (Maji et al., 2013). If nothing but expert annotations will suffice, data sharing lessens the burden on any one group. Multiple groups have used the International Skin Imaging Collaboration image set of human skin diseases (Codella et al., 2015; Haenssle et al., 2018) or the PlantVillage image set of plant diseases (Mohanty et al., 2016; Ghosal et al., 2018; Picon et al., 2019).

Identifying plant diseases *via* machine learning presents two challenges that limit the feasibility of the above solutions. First, qualified expert judgment is needed at some point in the annotation process, since there are often many causes for tissue death (e.g., disease, abiotic stress, physical damage, natural senescence) and the average person has no experience distinguishing among these. Second, there are hundreds of economically important plant diseases, each with unique considerations of host tissue appearance, plant architecture, symptomatology, etc. A group aiming to implement machine learning detection of a given disease for the first time will likely have to generate novel training data.

The identification of plant disease symptoms in an image might belong to one of three classes of tasks, per Liu et al. (2018): classification, detection, or segmentation. Object classification methods detect the presence or absence of features within an image on the whole, e.g., “this is an image of wheat stem rust.” Object detection methods identify the location and extent of symptoms within an image on a coarse spatial level, most commonly delineating them with bounding boxes. Semantic segmentation methods delineate the boundaries of features, assigning each pixel of an image to a given class, e.g., leaf, soil, or disease symptom. In this paper, we undertake this last task-identifying and outlining every diseased region in an image.

Aerial plant disease detection *via* machine learning has aroused much interest in the past few years, as evidenced in many reviews, letters, and prospectives (Araus and Cairns, 2014; Singh et al., 2016; Tsaftaris et al., 2016; Shakoor et al., 2017; Yang et al., 2017; Ubbens and Stavness, 2017; Chouhan et al., 2019; Maes and Steppe, 2019). Compared to the level of interest, relatively few examples have been published. Machine learning classification has been used to classify entire plants as virus-infected or not (Ha et al., 2017; Sugiura et al., 2018). Object detection methods have been used to identify diseased regions of grape plants (Kerkech et al., 2018) and diseased leaves of soybean (Tetila et al., 2017). Semantic segmentation of unmanned aerial vehicle (UAV) images, the task we undertake here, has been implemented in soybean (Tetila et al., 2019), tea plants (Gensheng et al., 2019), and maize (Stewart et al., 2019).

In the course of our previous work, we labeled over 100,000 examples of northern leaf blight (NLB), a fungal foliar disease of maize that causes gray-brown necrotic lesions (Wiesner-Hanks et al., 2018). Each of these annotations consisted of a line drawn down the principal axis of a lesion. With these line annotations, we trained convolutional neural networks (CNNs) to recognize NLB lesions in images taken by hand with 96.7% accuracy (DeChant et al., 2017) and in aerial field images with 95.0% accuracy (Wu et al., 2019). Delineating lesion boundaries with polygons would be ideal, as such annotations can ultimately yield much more precise image segmentation than lower-resolution annotations (Bell et al., 2015). Drawing such polygons is prohibitively time-consuming to do with only a small number of trained experts, however.

**Figure 1:**
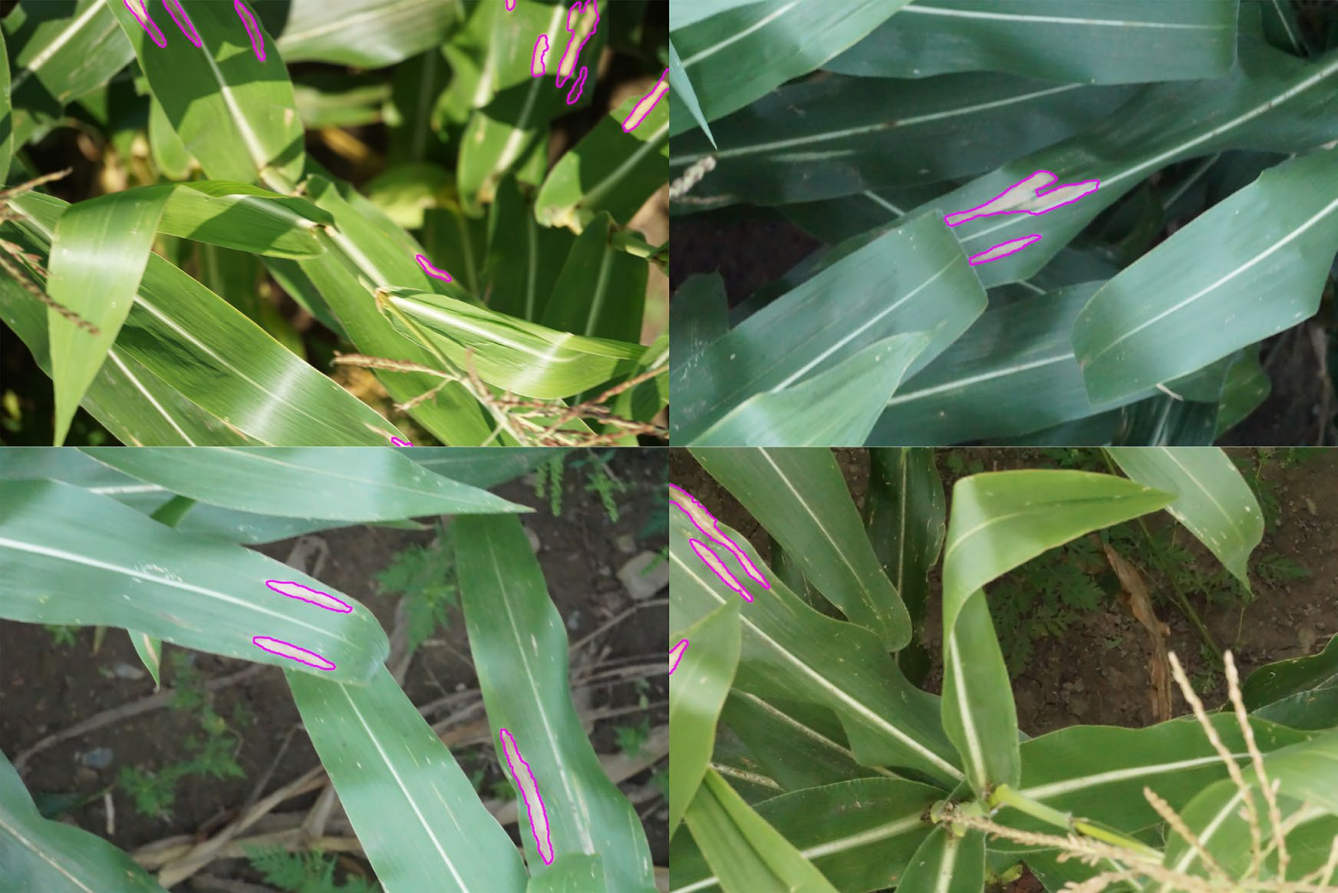
Examples of lesion segmentation on original images taken in the field by unmanned aerial vehicle. Regions classified as disease lesions by model outlined in magenta.

In this study we describe and implement a two-step approach for generating large amounts of high-resolution training data that has been vetted by qualified experts. First, experts identify disease symptoms, annotating them quickly at low resolution. Second, the more time-consuming task of annotating the lesion boundaries is outsourced to anonymous online workers through Amazon’s Mechanical Turk platform. This two-step approach allows us to maintain the reliability of expert diagnosis while also exploiting the speed and scale of crowdsourcing, producing a model with high accuracy and spatial resolution (**Figure 1**) with only one fifth as many expert-generated annotations.

## Materials and Methods

### Image Annotation

**Figure 2:**
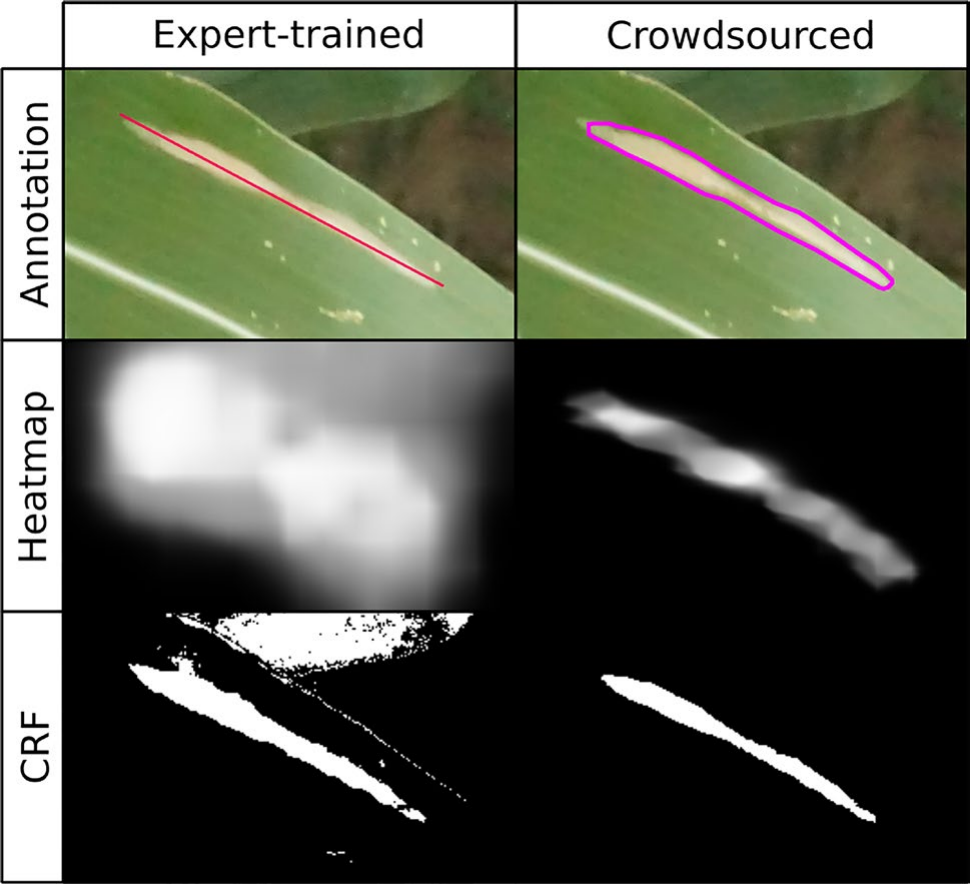
Comparison of annotations used and results of expert-drawn-lines model (left; Wu et al., 2019) and crowdsourced-polygon model described here (right). Top row: original image with annotations overlaid. Middle row: heatmap created by applying convolutional neural network in sliding window across image, brightness indicating probability of lesion at a given point (white = lesion, black = non-lesion). Bottom row: binary mask output of conditional random field segmentation using original image and heatmap.

All Mechanical Turk human intelligence tasks (HITs) consisted of one or more prompts to draw a single bounding polygon delineating the boundaries of a single lesion (**Figure 2**, top right), previously annotated with a line down the major axis by one of two human experts (Wiesner-Hanks et al., 2018). All images and annotations used, generated, or described herein are available in an Open Science Framework repository (https://osf.io/p67rz).

For each annotated lesion, a subimage was taken of the same width and height of the annotation line, plus 150 pixels padding on all four sides, so that workers had some context to the image. The annotation lines mostly spanned 400–1200 pixels in the x- and y-dimensions (depending on orientation), so this padding usually expanded the field of view by 25–75%. Workers were given basic instructions asking them to draw a polygon delineating the edges of the necrotic lesion with between 10 and 15 vertices, along with an example lesion thereof (**Supplemental text**, **Supplemental Figure 1**). The annotation lines drawn by experts were included in these subimages in red to make clear which lesion to annotate, as there was often more than one lesion in a single subimage.

HITs were deployed in three batches over the course of 2 months. Three unique workers were assigned to complete each HIT. Each worker was paid $0.03/HIT, an amount chosen to be similar to payment for comparable tasks deployed on Mechanical Turk at that time, adjusting for the fact that different HITs involve a different number of tasks. An additional $0.01 was paid to Amazon each time a worker completed a HIT, resulting in a total cost of $0.12/lesion (three workers per lesion, $0.03 per worker, $0.01 to Amazon per worker).

Annotations drawn by Amazon Mechanical Turk (MTurk) workers were first screened to see how much they agreed with the other annotations drawn on the same lesion. If a given worker drew polygons that rarely agreed with those drawn by other workers, their annotations were potentially suspect. After a batch was completed, the Intersection over Union (IoU), also called the Jaccard similarity, was calculated for each pair of polygons drawn on the same lesion by taking the area in pixels of their intersection divided by the area in pixels of their union. Each polygon was thus compared to the two other polygons drawn by other workers on the same lesion. If the mean Jaccard similarity between all annotations drawn by a given worker and those drawn by other workers was <0.5, the worker was flagged for manual review. This threshold was set at 0.5 because the vast majority of workers had overall mean IoUs in the 0.5–0.8 range, while a small number, who mostly completed only a handful of HITs each, had mean IoUs in the 0–0.5 range (**Supplemental Figure 2**). Manual review was deemed necessary, since a worker may have drawn their high-quality annotations compared to low-quality annotations. If their work was found to be unacceptable, all of their annotations were rejected and lesion subimages were redeployed as needed until three unique workers had acceptably annotated each. In all cases, workers whose annotations were rejected appeared to be drawing polygons at random.

The IoU was also used to filter out low-quality lesions. Entire UAV images were filtered automatically prior to annotation and manually during annotation, as described previously (Wiesner-Hanks et al., 2018), but individual lesions in an image could still vary in how clear and defined the visible symptoms were. Preliminary manual inspection of MTurk annotations revealed that lesions on which otherwise well-performing workers drew lesions with low overlap with one another were often blurry, ambiguous, or otherwise unacceptable. Only lesions for which all three polygon annotations had an IoU >0.6 with one another (a threshold chosen to filter out roughly the bottom 25% of lesions) were used to generate images for model training as described below. The mean and standard deviation of pixel red, green, and blue (RGB) values, used for later normalization of images, were calculated on these whole images.

Training, validation, and test data were generated based on the method used with polygon annotations in the OpenSurface dataset (Bell et al., 2015). Multiple square subimages, hereafter referred to as “patches,” were cropped from the entire UAV image and classified as “lesion” or “non-lesion” based on whether the exact center point of the image lay within a lesion. To generate positive patches (the “lesion” class), pixels lying within at least two of three annotation polygons were used as a search space. From these, random points were sampled *via* Poisson-disk subsampling (scipython.com/blog/poisson-disc-sampling-in-python/), with minimum distance of 200 pixels between each point. Negative patches (the “non-lesion” category), were chosen by randomly sampling points from the pixels in each image that were not included in any of the annotation polygons. Negative training images thus could contain a lesion, so long as they were not centered on one.

Because the original UAV images consisted mostly of non-lesion area, many more non-lesion patches could be extracted from the images than lesion patches. Preliminary model testing with sample images suggested that using a balanced dataset with an equal number of lesion and non-lesion patches biased model predictions toward false positives, i.e., detecting lesions where there were none (data not shown). We thus used a moderately unbalanced dataset and accounted for the class imbalance using weights in the loss function, as described below.

In order to make the model more generalizable, training images were augmented *via* random transformations that preserved the image class, i.e., the location of the central pixel in a lesion or not. Images were horizontally and/or vertically flipped, rotated by 0 to 90° either clockwise or counterclockwise, and scaled between 0.75x and 1.33x. As these images were taken from directly overhead, there was no need to preserve image orientation.

### Network Construction

We used a ResNet34 model (He et al., 2016) that had been pre-trained on the ImageNet dataset of several million labeled images (Russakovsky et al., 2015) as a generalized feature extractor, replacing the final fully connected layer with a fully connected layer of output dimension 2. The output tensor for each input image was a two-dimensional vector of scores for the two classes: centered on a lesion or not centered on a lesion (note that images containing a lesion but not exactly centered on it belong to this second class). A weighted cross-entropy loss function was used, which normalizes the scores into estimated probabilities *via* the softmax function, then takes the negative log of these probabilities and multiplied by the class weights to account for class imbalance. Class weights of 0.36 and 1.0 were used for lesion and non-lesion images, proportional to the number of images in each class.

In order to determine which patch size and learning rate was most appropriate, we analyzed performance on a smaller sample set of images. For both image classes (lesion and no lesion), 5% of the training and validation sets were randomly sampled. The above network was trained and validated on this 5% subsample with six patch sizes (square patches of size 200, 400, 500, 600, 800, or 1,000 pixels, using the same centerpoints for each size) and seven learning rates (1e−5, 3e−5, 1e−4, 3e−4, 1e−3, 3e−3, and 1e−2). With each combination of patch size and learning rate, the network was trained for 10 epochs with a step size of 10 and gamma of 0.1, corresponding to a 10-fold decrease in the initial learning rate every 10 epochs.

The best-performing parameters were then used to train the network on the entire training and validation set for 20 epochs with a step size of 10 and gamma of 0.1. Patches were resized to 224 by 224 pixels and treated with a random horizontal flip, then normalized using the previously calculated mean and standard deviation of pixel RGB values. To compare learning rate dropouts, the model was also trained using step sizes of 5 and 20, maintaining a gamma of 0.1. Weights were optimized using stochastic gradient descent with weights of 1.0 and 0.36 for the non-lesion and lesion labels, respectively, proportional to the number of images in each category. All training was done on an Nvidia GTX 1070 Ti GPU with batch size 120, randomizing image input order.

To visualize the model-estimated probability of a given region containing a lesion or not, heatmaps were generated by applying the final CNN on a sliding window across whole UAV images, then applying softmax transformation to generate probabilities for the two classes (centered on a lesion or not). To account for varying lesion sizes, we used the resizing approach of Bell et al. (2015). The image was resized by three separate scaling factors: the original scale *r* used in model training (such that a 500x500 window was resized to 224x224 pixels), r*sqrt(2), and *r*/sqrt(2). At these scales, a window of size 500x500, 690x690, or 345x345 pixels, respectively, mapped to 224x224 pixels. Images were padded on all sides *via* reflectance padding, and the trained model was applied *via* a sliding window approach across the entire image with a stride of 50 pixels in both dimensions. The resultant output was then resized to the original 4,000x6,000 pixels *via* bilinear interpolation. The three resultant heatmaps were then averaged, and this averaged heatmap was used for downstream analyses. For comparison, the trained model described by Wu et al. (2019) was applied in an identical manner. As the scaling used for training purposes was identical between these two models, the same scales were used for heatmap generation.

### Image Segmentation

Pixel-wise classification was performed using the fully connected conditional random field (CRF) method of Krähenbühl and Koltun, (2011), implemented in Python *via* pydensecrf. CRF optimization was performed using three separate color spaces: the original, untransformed RGB values, RGB values transformed to maximize contrast between lesion and non-lesion pixel values, and L*a*b* color space. For the second method, the pixels surrounding each polygon annotation were found by dilating the polygon mask (expanding the mask along its edges to include pixels for which a kernel overlaps with the mask) for five iterations using a 20 pixel by 20 pixel square kernel, then subtracting the area created by performing only one dilation of the mask. The RGB values of pixels within these regions and those lying within polygon annotations were then downsampled by a factor of 10 and analyzed *via* linear discriminant analysis (LDA) to obtain a transformation maximizing between-group differences in Euclidean distance between values in the two regions. RGB to L*a*b* transformation was performed using OpenCV, producing 0–255 integer-valued L*a*b* coordinates.

CRF performance is controlled the θ parameters, which determine how strongly pixel classification is influenced by proximity (is it close to many pixels believed to be NLB lesions)? and color (is it the same color as pixels believed to be NLB lesions)?. Because optimizing these is difficult (Krähenbühl and Koltun, 2011), we used a simple grid search to find suitable parameters, evaluating CRF performance for all combinations of θ values on a set of 118 training images. These were selected from the entire set of training images by choosing images in which the annotation polygons of all three workers agreed fairly well (each one having IoU > 0.8 with the union of all three, a cutoff chosen to be fairly stringent) for all lesions in the image. CRF performance on each image was evaluated under each color space with slightly different parameters, as appropriate for each. For the RGB and LDA-transformed color spaces, the kernel width θ_α_, corresponding to the spatial dimension of pixel correlation and deviation, was evaluated at values ranging from 10 to 600 by a step size of 10. For the untransformed RGB color space, θ_β_, corresponding to the color-space correlation and deviation of pixels, was evaluated at values ranging from 1 to 40, step size 1. For the LDA-transformed RGB values, θ_β_ was evaluated at values ranging from 0.1 to 0.4, step size 0.1. For the L*a*b* color space, separate kernel widths were used for the distance along the L dimensions and distance in a–b dimensions. CRF performance was analyzed for θ_α_ (still the spatial kernel width, unrelated to the a* color dimension) ranging from 10 to 500 with step size 10, θ_L_ ranging from 1 to 25 with step size 1, and θ_ab_ from 1 to 20 with step size 1. CRF performance on the model of Wu et al. (2019) was tested only in the RGB color space.

## Results

### Mechanical Turk Annotations

MTurk workers drew 15,240 polygon annotations on 5,080 lesions, cropped from 752 parent images collected by the UAV. Training data for the CNN were generated only from those images in which, for all lesions in the image, all three polygon annotations had an IoU of at least 0.6 with one another, leaving us with 588 UAV images containing 3,834 annotated lesions. Poisson-disk subsampling of the lesion polygon annotations yielded 22,193 centerpoints that were used to generate 22,193 positive images (**Table 1**). From the same 588 UAV images, we sampled 58,800 negative images, 100 from each image. Both positive (centered on a lesion) and negative (not centered on a lesion) images were divided into training, validation, and test sets in a 70:15:15 ratio.

**Table 1:** Number of images sampled of each label (lesion *vs.* no lesion) and their division into training, validation, and test sets.

Phase	Number of images	
	Lesion	No lesion
Training	14,783	41,160
Validation	3,168	8,820
Test	3,168	8,820

**Figure 3:**
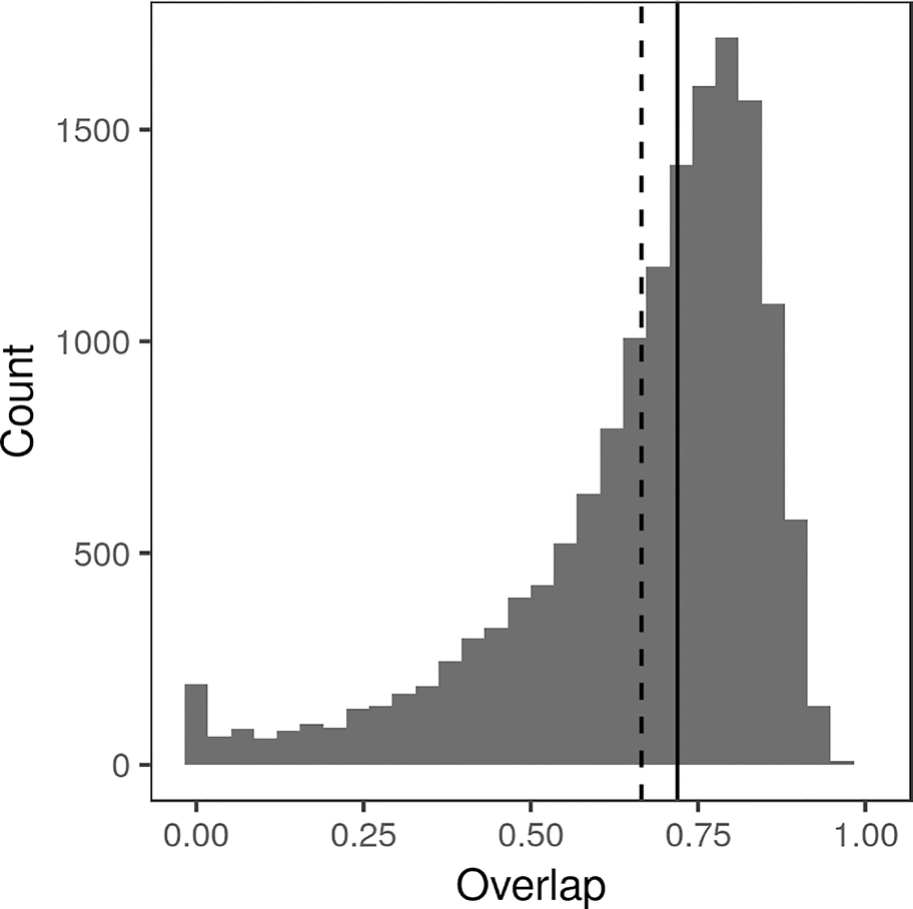
Histogram of Intersection over Union (IoU) between all pairs of polygon annotations drawn by Amazon Mechanical Turk workers, calculated as the area in pixels of intersection divided by the area in pixels of the union. Median IoU (0.7265) indicated by solid line, mean IoU (0.6832) indicated by dotted line.

Most workers annotated only a few images, with a small number of workers annotating several hundred (**Supplemental Figure 3**). On average, it took an MTurk worker 32 s to annotate a single lesion (median 27 s, standard deviation 19 s). All sets of deployed HITs were fully annotated in under 2 h. Workers generally performed fairly well, as shown by the fact that any two annotations drawn on the same lesion tended to overlap (**Figure 3**). Most pairs of polygons (83.2%) had an IoU of at least 50%. Manual examination found that many of the annotations with low IoU were on images that were blurry, ambiguous, or otherwise undesirable. Workers were paid $0.03/lesion, resulting in an average payment of only $3.75/h for annotation.

### Model Performance

**Figure 4:**
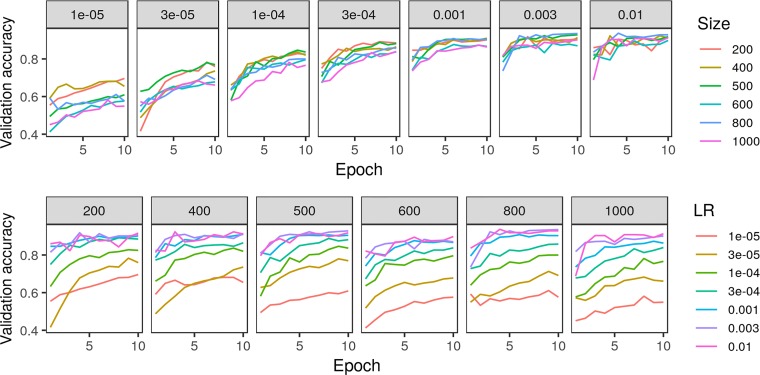
Comparison of crowdsourced convolutional neural network accuracy on 5% subsample of training/validation images across various parameters of learning rate (LR) and patch size in pixels.

Testing classification accuracy of the crowdsourced CNN on a subsample of training and validation images, we found a learning rate of 3e−3 and a patch size of either 500 or 800 to be best (**Figure 4**). Though classification accuracy was slightly higher when using a patch size of 800 compared to a patch size of 500, we chose a patch size of 500 to be consistent with that used in the model trained on expert-drawn-lines (Wu et al. in press) to facilitate comparisons between the two.

**Figure 5:**
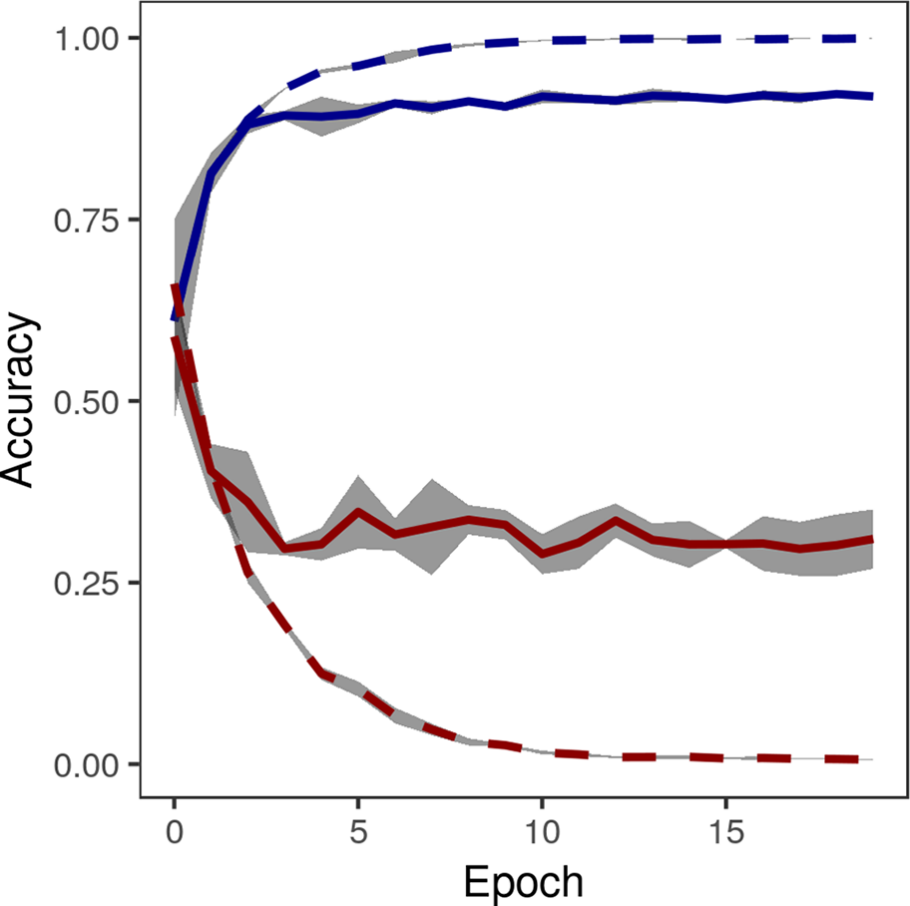
Accuracy (blue) and loss (red) of convolutional neural network on training images (dashed lines) and validation images (solid lines) converged by 15 epochs. Gray area shows standard deviation of accuracy over five replications of training on the same training/validation sets.

The accuracy of the crowdsourced CNN on the validation set of image crops converged by 15 epochs (**Figure 5**). One concern with any machine learning model is the possibility of overfitting: training a model that performs well on the specific data set being used, but that is not generalizable and performs poorly on new data. Loss on the validation set did not tend to increase after reaching a global minimum, suggesting that overfitting was not a major concern (James et al., 2017), though the gap between training loss and validation loss suggested some overfitting (**Figure 5**). On the final held-out test set of image crops, the crowdsourced CNN performed well, achieving an overall classification accuracy of 0.9741, precision [TP/(TP+FP)] of 0.9351, recall [TP/(TP+FN)] of 0.9694, and F1 (harmonic mean of precision and recall) of 0.9520 (**Table 2**).

**Table 2:** Predictions of the final network on the held-out test set.

Prediction	Image	
	Lesion	No lesion
Lesion	3,071	213
No lesion	97	8,607

### Image Segmentation

Applying a fully connected CRF to the heatmaps generated by the crowdsourced CNN and the held-out test images, we were able to accurately classify each pixel of an image as lesion or non-lesion with high spatial resolution (**Figure 2**, bottom row). Pixel-wise classification accuracy was high even when heatmaps were clearly not suitable, as the vast majority of most images is non-lesion, so a model that classified all pixels as non-lesion would still achieve an accuracy of 0.9940. For this reason, F1 was taken to be a more suitable metric for image segmentation performance than accuracy.

**Figure 6:**
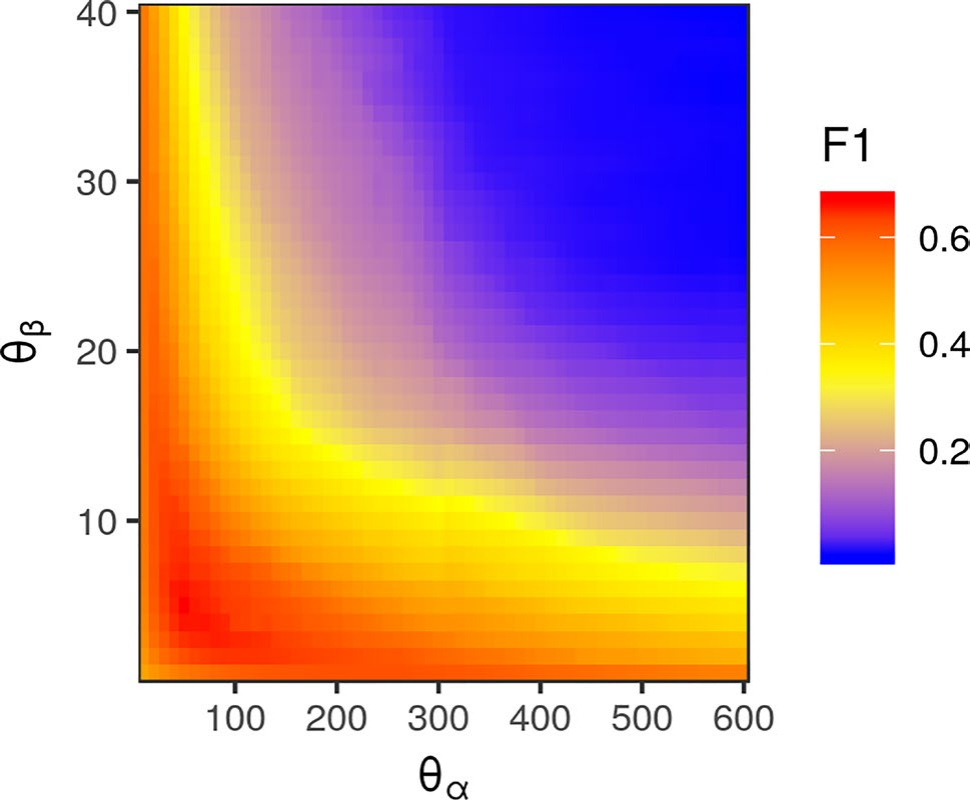
Heatmap of pixel-wise F1 score of conditional random field (CRF) segmentation across different levels of θ_α_, corresponding to the spatial scale of correlations between pixel color values, and θ_β_, corresponding to the color space scale of correlations. Values were determined using images transformed with red, green, and blue values transformed *via* linear discriminant analysis-derived differentiation transformation, as this was the color space in which CRF segmentation performed best.

**Figure 7:**
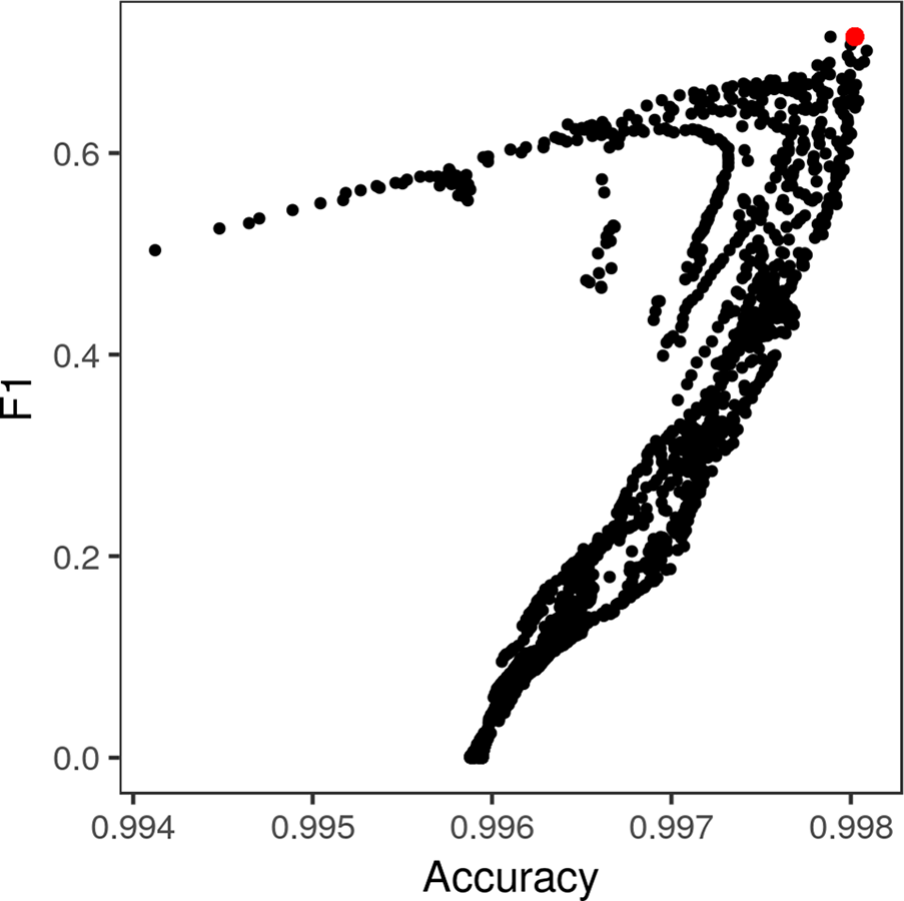
Pixel-wise F1 score of lesion/non-lesion segmentation *vs.* accuracy thereof across different levels of θ_α_ and θ_β_. The conditional random field parameters that yielded the highest F1 score (red point) also yielded near-maximum accuracy of segmentation. Each point represents a single combination of θ_α_ and θ_β_ tested in the grid search (**Figure 5**).

Exhaustive grid search found the best-performing θ parameters for each color space to be θ_α_ = 50, θ_β_ = 5 for the standard RGB color space, θ_α_ = 110, θ_L_ = 25, and θ_ab_ = 1 for the L*a*b* color space, and θ_α_ = 70 and θ_β_ = 0.7 for the LDA-transformed color space (**Figure 6**). Transforming images into the L*a*b* color space moderately increased segmentation accuracy. The best-performing CRF parameters segmented images with an accuracy of 0.9957 and F1 of 0.6695 in the RGB color space, compared to peak accuracy of 0.9977 and F1 of 0.6777 in the L*a*b* color space. Transforming the RGB values using the matrices obtained *via* LDA was the most effective, yielding a peak accuracy of 0.9981 and F1 of 0.7153. The parameters that segmented LDA-transformed images with the highest F1 score also did so with near-maximum accuracy (**Figure 7**).

**Figure 8:**
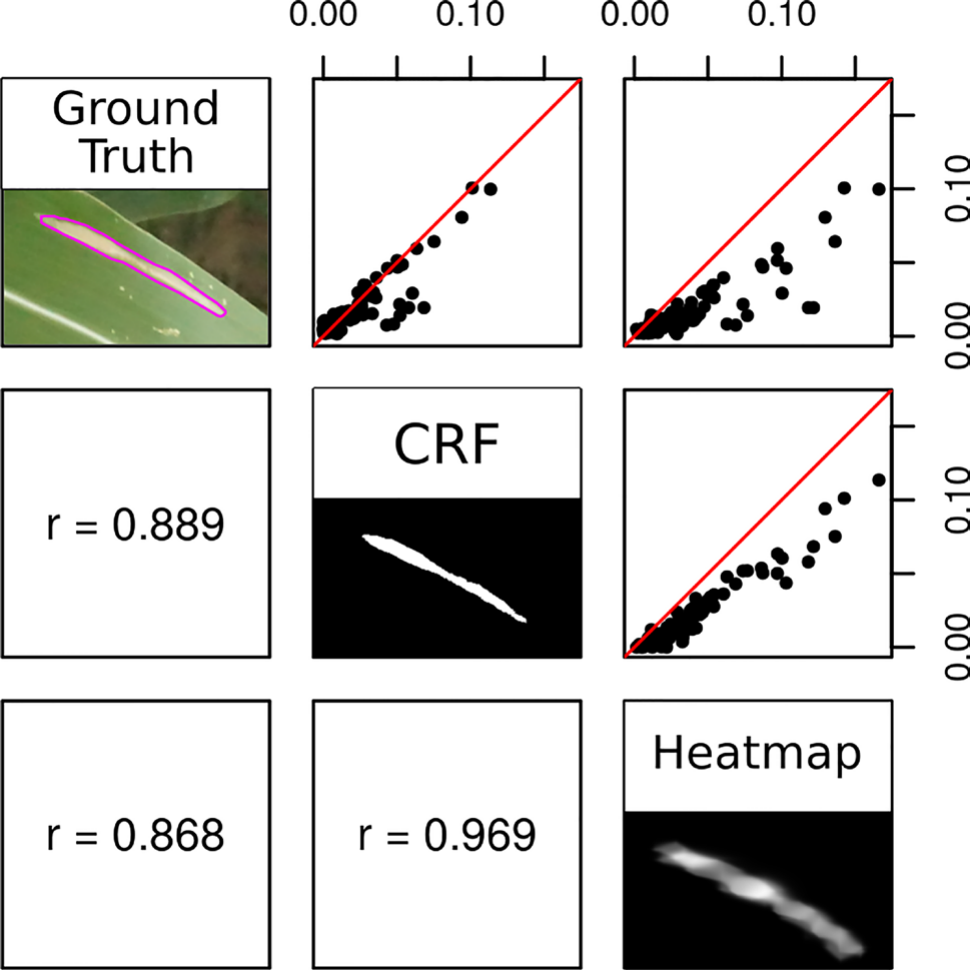
Correlation between the proportion of a test image classified as lesion in ground truth (consensus polygons of three high-quality Amazon Mechanical Turk annotations), conditional random field segmentation, and heatmap thresholded at 0.5. Red lines depict 1:1 ratio.

CRF segmentations could be used to accurately estimate the proportion of an image covered by lesions (**Figure 8**). The proportional lesion coverage estimated by CRF was highly correlated to ground truth estimates. The heatmaps themselves could also be used to estimate proportional lesion coverage in an image, bypassing the CRF step. Thresholding probability heatmaps at 0.5 produced binary images, in which pixels had a value of 1 if the interpolated predicted softmax probability of the “lesion” prediction was higher and a value of 0 if that of “non-lesion” was higher. However, the lesion coverages estimated by CRF segmentation were proportional to the ground truth areas in an approximately 1:1 manner, while the areas generated from thresholding probability heatmaps were artificially inflated (**Figure 8**).

**Figure 9:**
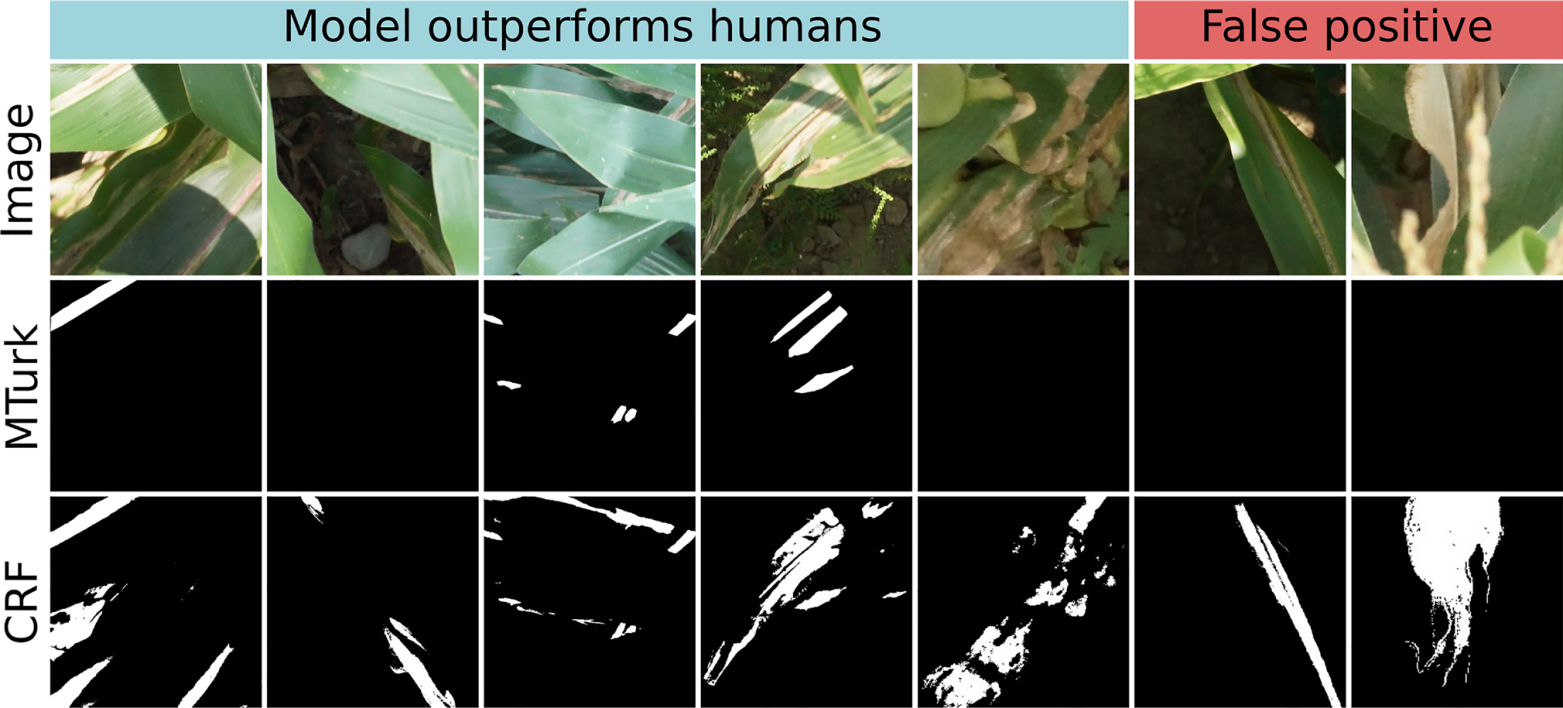
Original image (top row), ground truth annotations drawn by Amazon Mechanical Turk workers (middle row), and conditional random field (CRF) segmentation (bottom row) for all seven test images in which CRF segmentation and ground truth diverged highly. In left five images, crowdsourced-polygon model outperformed humans by identifying lesionated areas where human experts had missed them. In the right two images, the model falsely classified senescent leaf tissue as lesions. White = lesion, black = non-lesion.

Image segmentation using the crowdsourced CNN and CRF tended to outperform human experts. There were seven instances in which the proportion of a test image classified as “lesion” diverged highly between CRF segmentation and ground truth (**Figure 8**, outliers lying off of the red 1:1 line). This was surprising, as precision of CRF segmentation was higher than recall (0.7388 *vs.* 0.6937) on a pixel-wise basis. Examining these seven cases more closely, we found that five of them were due to the model correctly locating lesions missed by the experts, while only two were due to the model misidentifying senescent leaves as lesions (**Figure 9**). Excluding the five images in which the CRF outperformed human experts, the Pearson’s correlation between the proportion of pixels in an image labeled as lesions in the ground truth masks and the proportion classified as lesions by the CRF segmentation rose from 0.8893 to 0.9428. Thus, while there is room to improve the model by addressing false positives, it was more often than not outperforming trained human experts.

The two-step image segmentation process was fairly slow, however. Heatmap construction by the sliding-window approach using three different scales took a mean of 38.1 s on a 4,000x6,000 image: 10.8 s at original scale *r*, 6.2 s at scale *r*/sqrt(2), and 21.1 s at scale r*sqrt(2). CRF segmentation of a 4,000x6,000 image took 2.8 s on average. Newer end-to-end segmentation methods should be able to improve on this, as discussed below.

### Benefits of Crowdsourcing

Using crowdsourced polygon annotations greatly improved the spatial resolution of the final model with far less time investment from experts. We compared two CNNs of similar structure and implementation: one trained on lines drawn by experts (Wu et al., 2019) and the one trained on crowdsourced polygons, described here. These models were used to perform semantic segmentation using the same approach, *via* applying the CNN on a sliding window across images to generate probability heatmaps, then feeding these heatmaps into an optimized CRF to perform the final segmentation. Using the same approach with both model outputs isolated the effects of using the more information-rich crowdsourced polygons, rather than differences in segmentation methods.

**Figure 10:**
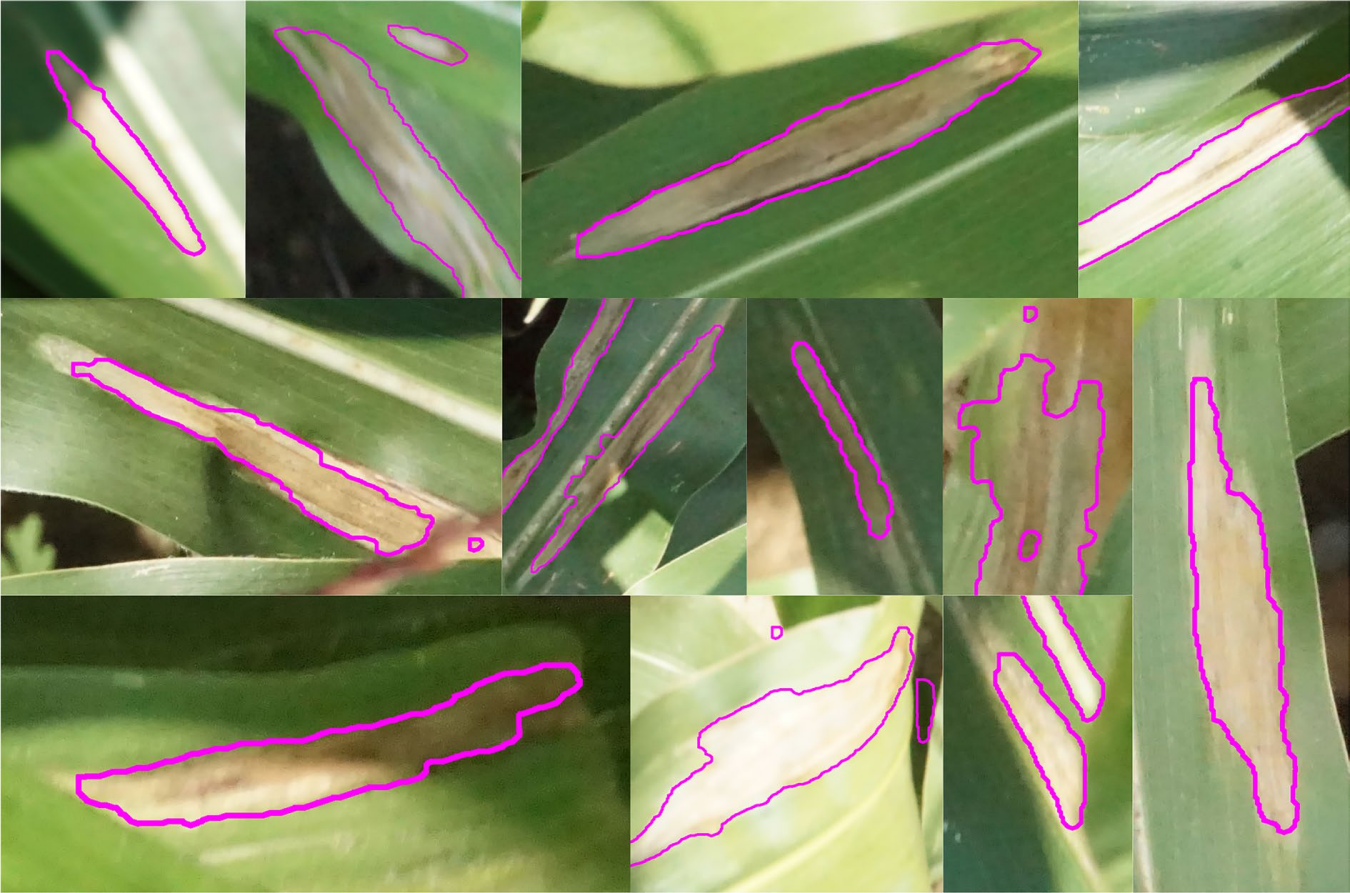
Close-up view of image segmentations performed by conditional random field using heatmaps generated by the crowdsourced convolutional neural network. Magenta outline shows lesion boundaries from 12 randomly selected images in the test set.

Using the crowdsourced annotations provided three key benefits. First, the greater spatial resolution of polygon annotations allowed us to reliably delineate individual lesions with millimeter-level accuracy (**Figure 10**), which could not be done with line annotations alone. CRF segmentation using the crowdsourced CNN output was able to segment images into lesion and non-lesion pixels with a maximum F1 of 0.76 on the validation image set, while segmentation using expert-drawn lines achieved a maximum F1 of only 0.21.

Second, the crowdsourced-polygon model was able to achieve this higher spatial resolution using only one-fifth as many annotations. The crowdsourced CNN was trained, validated, and tested on only 5,080 expert-drawn lesions, compared to the 25,508 used for the expert-drawn-lines model (Wu et al., 2019).

Third, crowdsourcing allowed us to generate these polygons more quickly than would be possible using only a handful of experts. Drawing a line took far less time than drawing a polygon. Examining the timestamps of the annotations, we found that experts took 4.38 s on average to annotate a lesion with a line, while it took an MTurk worker a mean of 32 s to draw a polygon. An expert could thus annotate 1,000 lesions with lines in 73 min on average, while a non-expert would take 533 min to annotate 1,000 lesions with polygons. The more complex non-expert task thus took 7.3x more time than the simpler expert task. Due to the parallel nature of crowdsourcing, however, all 5,080 lesions were annotated by MTurk workers in less than 15 h.

A comparison of the total time needed to generate training data at the scale used in this study shows the benefits of a two-step crowdsourcing approach. A single human expert can annotate 5,000 lesions in roughly 6 h, which could then be completely annotated with polygons by MTurk workers in one to 2 days. Assuming this expert worked as fast as the average MTurk worker (including locating lesions, which MTurk workers were not required to do), drawing these polygons would take them roughly 44 h. Crowdsourcing the more laborious part of the task as described here is a more efficient use of plant scientists’ time and expertise.

## Discussion

Our full method, combining a CNN applied across a sliding window and image segmentation *via* a fully connected CRF, was able to identify and delineate disease lesions at the millimeter level, the smallest spatial scale reported so far for aerial plant disease detection. The two-step approach for generating training data, in which experts annotate symptoms in low detail and non-experts annotate them further in high detail, was critical to achieve high spatial resolution. Without the non-expert polygon annotations, our previous effort was able to identify lesions with high accuracy at a sub-leaf scale (Wu et al., 2019), but not at sufficient resolution to accurately segment an image and delineate individual lesions. With them, we were able to segment images down to the millimeter with sensitivity surpassing that of human experts: in five out of seven cases in which human ground truth and model predictions diverged, the model had correctly identified disease symptoms where experts had missed them (**Figure 9**).

Using Mechanical Turk, thousands of images could be annotated in only a few hours, reducing what was until then a major bottleneck in the model training process. Despite the fact that these workers (presumably) have no experience in plant disease diagnosis, their annotations were generally of high quality and could be used to train the model without the need for an expert to look over each one. With three annotations for each image, we were able to identify and filter both low-performing workers, whose annotations tended not to agree with others, and low-quality images, on which multiple (otherwise well-performing) workers drew annotations that did not agree. There are several possibilities for improving the MTurk annotation process. Increasing the number of workers per image could increase the quality of annotation polygons or the ease of identifying low-quality images.

The cost of crowdsourcing *via* MTurk was quite low, at $0.03/lesion, implying a wage of $3.75/h based on the average time to annotate a lesion. Future studies would ideally compare different payment structures in order to maximize worker payment, minimize overhead, and maintain or increase annotation quality. Restructuring the HIT so that each consists of annotating multiple lesions, rather than just a single lesion, would decrease the payment to Amazon per image while paying workers the same per HIT. Many HITs posted on MTurk require a short qualification test to vet workers. In our case, workers could be asked to annotate three lesions adequately in order to be approved to complete HITs. Increasing worker payment in tandem with this could attract and retain better-performing annotators, providing workers with a higher wage while decreasing the amount of post-processing needed to filter out low-confidence annotations.

We used a two-step method for semantic segmentation, first training a model to classify lesions, then using a sliding window approach and CRF to turn these classifications into semantic segmentation of a full image. This allowed us to make a useful comparison to a model trained on coarse, expert-generated annotations, since the same segmentation method could be used with both models’ output, isolating the impact of the annotation data rather than the segmentation approach used. However, newer methods for semantic segmentation, such as region proposal networks (Ren et al., 2016) or atrous convolution (Chen et al., 2017) might well perform the task better and faster.

A chief limitation of this method is the difficulty of acquiring field images at high enough resolution and clarity such that individual lesions can be discerned. Capturing images in which each pixel represented a millimeter or less at canopy level required slow flights at low altitude with a high-zoom lens (Wiesner-Hanks et al., 2018), not ideal for comprehensively imaging a large area. This challenge would be even greater when working with a disease with small or inconspicuous symptoms—chlorosis, leaf curling, lesions only a few millimeters in diameter—as opposed to the large, obvious lesions of NLB. Targeted sampling of a field, rather than attempting to image every plant, can still give growers a large amount of information with which to make decisions regarding disease management. Acquiring images and diagnosing lesions every 10 m or so would only analyze a very small proportion of a field’s total area, but it would provide much more information compared to the zig-zag walking paths commonly used when scouting for pests and diseases (Doll et al., 2016).

UAVs are now a common part of many US growers’ field operations, and interest continues to grow (Luck et al., 2018; Miller and Adkins 2018; Purdue Extension Annual Report 2018). The use of UAVs for disease diagnosis is still in its infancy, however. We predict that UAV-based disease phenotyping will be most readily adopted in crops with a high value per acre where fungicide usage is common, such as grapes or almonds. In such crops, the added benefit of fast, frequent, reliable disease screening is most likely to outweigh the time and monetary costs needed to develop the diagnostic platform. As UAV and imaging technology progress, and more and more image datasets are generated and freely shared among researchers, we believe that UAV-based deep learning will become simpler to implement and will soon be a useful tool for growers and geneticists across many crops and pathosystems.

## Data Availability Statement

All images and annotations used, generated, or described herein are available in an Open Science Framework repository (https://osf.io/p67rz).

## Author Contributions

Author TW-H planned and executed the crowdsourcing project, developed the models used to process the crowdsourced data, and wrote the manuscript. Authors ES and NK developed the UAV platform and captured the images. Authors TW-H and ES created the expert annotations. Authors HW and CD advised on CNN development and implementation. Authors HL, MG, and RN were PIs on the grant that funded this work and provided top-level project planning. All authors contributed to project design and editing of the manuscript.

## Funding

This research was funded by the U.S. National Science Foundation National Robotics Initiative (grant number 1527232).

## Conflict of Interest

Author TW-H was employed by company PepsiCo during preparation of this manuscript.

The remaining authors declare that the research was conducted in the absence of any commercial or financial relationships that could be construed as a potential conflict of interest.
