# Supplementary material for: Millimeter-Level Plant Disease Detection From Aerial Photographs via Deep Learning and Crowdsourced Data
Source: Front Plant Sci. 2019 Dec 12;10:1550. doi: 10.3389/fpls.2019.01550 (PMC6927297; doi:10.3389/fpls.2019.01550)
Supplement: Supplementary file 1 [file DataSheet_1.docx]

**SUPPLEMENTARY MATERIAL**

**Supplemental text: worker instructions**

Place points around the edge of the lesion, where the dead brown tissue meets the healthy green tissue, as shown in this example. There may be multiple lesions in the image- only label the one with a red line down the center.

You must place at least 10 points, but no more than 15. When done placing points, right click to close the polygon. *Once you close a polygon, you cannot delete the most recent point*.

To move a point, click "Edit", then drag the point. To delete a polygon, click "Edit", select it, then click "Delete Selected Polygon".

If the image is very blurry, please draw the polygon as best you can and place a comment (e.g. "blurry") in the box below, then submit.

**
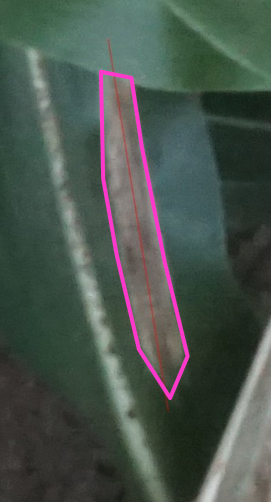
**

**Supplemental Figure 1**. Example lesion annotation shown to MTurk workers.

**
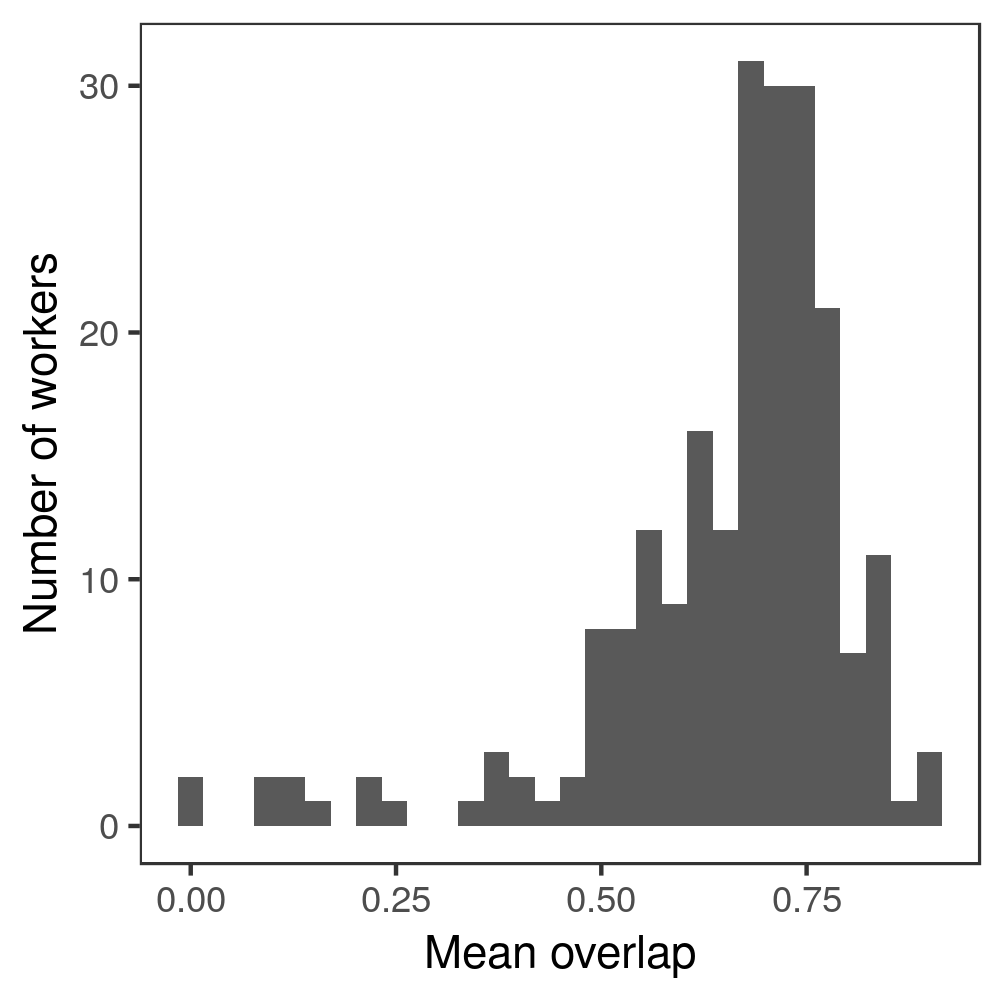
**

**Supplemental Figure 2**. Histogram of mean overlap (Intersection over Union) of all polygons drawn by a worker.

**
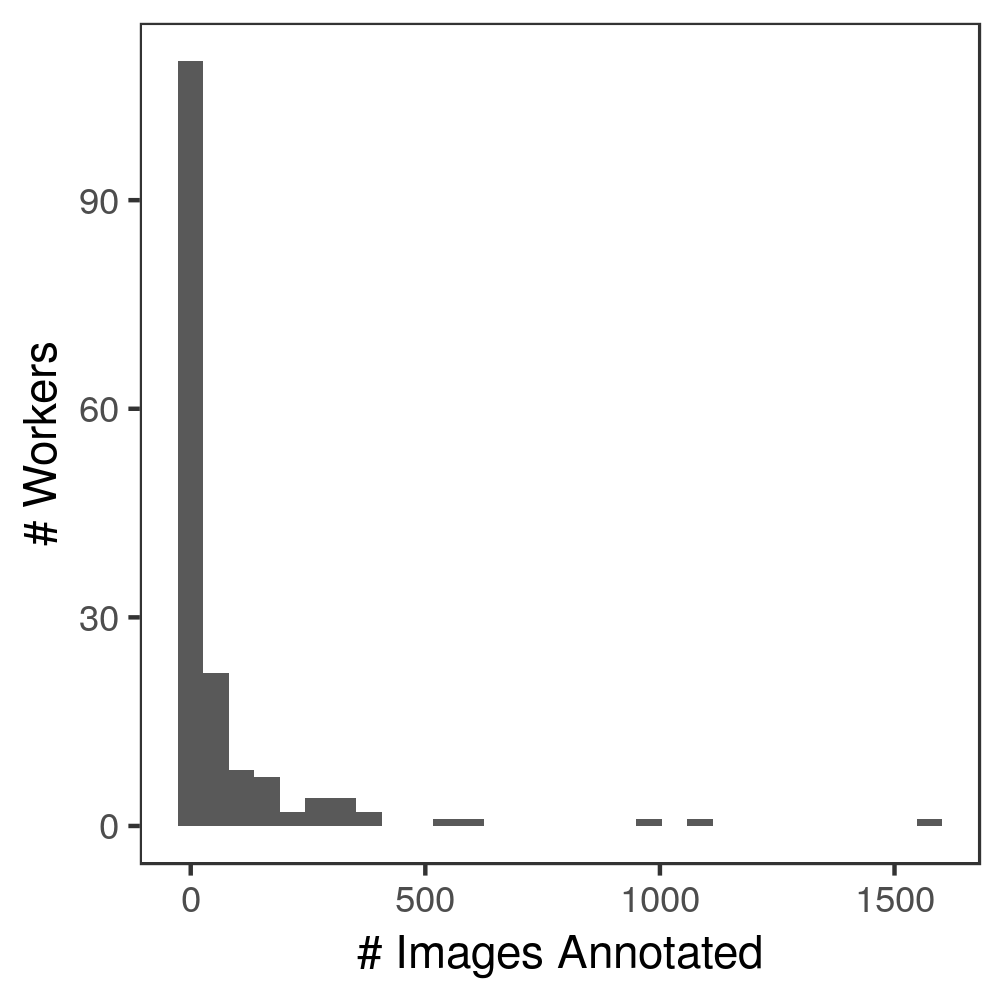
**

**Supplemental Figure 3**. Histogram of number of lesions annotated by individual MTurk workers.
